# A monthly temperature prediction based on the CEEMDAN–BO–BiLSTM coupled model

**DOI:** 10.1038/s41598-024-51524-7

**Published:** 2024-01-08

**Authors:** Xianqi Zhang, He Ren, Jiawen Liu, Yuehan Zhang, Wanhui Cheng

**Affiliations:** 1 Water Conservancy College, North China University of Water Resources and Electric Power, Zhengzhou, 450046 China; 2 Collaborative Innovation Center of Water Resources Efficient Utilization and Protection Engineering, Zhengzhou, 450046 China; 3 Technology Research Center of Water Conservancy and Marine Traffic Engineering, Zhengzhou, 450046 Henan Province China

**Keywords:** Environmental impact, Climate sciences

## Abstract

Temperature as an important indicator of climate change, accurate temperature prediction has important guidance and application value for agricultural production, energy management and disaster warning. Based on the advantages of CEEMDAN model in effectively extracting the time–frequency characteristics of nonlinear and nonsmooth signals, BO algorithm in optimizing the objective function within a limited number of iterations, and BiLSTM model in revealing the connection between the current data, the previous data and the future data, a monthly average temperature prediction model based on CEEMDAN–BO–BiLSTM is established. A CEEMDAN–BO–BiLSTM-based monthly average temperature prediction model is developed and applied to the prediction of monthly average temperature in Jinan City, Shandong Province. The results show that the constructed monthly mean temperature prediction model based on CEEMDAN–BO–BiLSTM is feasible; the constructed CEEMDAN–BO–BiLSTM model has an average absolute error of 1.17, a root mean square error of 1.43, an average absolute percentage error of 0.31%, which is better than CEEMDAN–BiLSTM, EMD–BiLSTM, and BiLSTM models in terms of prediction accuracy and shows better adaptability; the constructed CEEMDAN–BO–BiLSTM model illustrates that the model is not over-modeled and adds complexity using Friedman’s test and performance comparisons between model run speeds. The model provides insights for effective forecasting of monthly mean temperatures.

## Introduction

Since the twenty-first century, global warming has become a global shared problem, the most important of which is the change of temperature, which is closely related to the healthy life of human beings, the economic development of society and other activities^1^. Accurate temperature prediction plays an important role in people's living arrangement and health as well as agricultural food supply, and also provides important basis and data support for energy management, disaster warning and disaster prevention and mitigation. However, temperature series are affected by various aspects such as atmospheric circulation, monsoon climate, etc., and their time series data have obvious randomness and uncertainty. Scholars at home and abroad have carried out a lot of research on temperature prediction^2^, Karevan et al.^3^ established the transformed long-short memory network model (T-LSTM) on the basis of the long-short memory network model (LSTM) to predict the weather, and the performance of the T-LSTM model is better than the LSTM model in practical applications; Karimi et al.^4^ constructed a monthly temperature based on the RF prediction model and used monthly temperature observations from 30 different weather stations in Iran between 1986 and 2000 as an example, and used three different performance evaluation indexes to conduct a comparative study of the traditional SVM model, and the results of the study showed that the RF model had better prediction results than the SVM model; Lee et al.^5^ utilized the EMD approach to the phenomenon of non-stationary oscillations in climate research and proposed a non-stationary oscillation resampling model. The results of simulation experiments showed that the model was able to provide useful forecasts of future series using the long-term oscillation patterns of the observed data^5^; Yakut et al.^6^ constructed a method for forecasting monthly mean temperature based on an ANN model, an adaptive neuro-fuzzy inference system, and an SVR model, which further improved the accuracy of forecasting the monthly mean temperature in Turkey; Mohammadi et al. utilized a linear regression time series model to calculate the minimum, maximum, and mean temperatures. They also created a novel hybrid model by combining AR and nonlinear time series models. This hybrid model is composed of MLP-AR, MLP-ARARCH, and AR-ARCH models^7^; Chithra et al. employed a feed-forward back-propagation neural network model to forecast the monthly maximum and monthly minimum temperatures within the Chaliyar river basin in Kerala, India. The purpose of their study was to identify climate change patterns or signals^8^. Hausfather et al.^9^ analyzed the performance of climate models published between 1970 and 2007 in predicting future changes in global mean surface temperature (GMST) and found that the proposed models all show warming consistent with the observed structure. This was later confirmed by Kay in Nature. Wang et al.^10^ introduced and assessed a novel algorithm utilizing pattern approximation matching for outdoor temperature prediction. The algorithm leveraged historical data from five Chinese cities, representing distinct climatic regions. By optimizing the parameters using the historical data of each city, the algorithm's effectiveness in forecasting outdoor air temperatures was evaluated. The accuracy of the predictions was verified through a comparison of the predicted values with the measured outdoor air temperatures^10^. Zhang et al. used a stacked long- and short-term memory network model (stacked LSTM) to predict the air temperatures in the next half hour from historical observations. Through a comparison with the deep neural network (DNN) and random forest (RF) methods using different sliding windows, it was determined that the stacked LSTM network outperformed both the DNN and RF methods^11^. Huang et al. proposed a new nonlinear target prediction method based on RNN to predict the daily average, maximum and minimum temperature data of 14 stations for each site's daily maximum and daily minimum temperatures for the next 24 h. The 24 h short-term forecasts were also validated during the modeling process. The prediction outcomes of the RNN model were compared to those of the stepwise regression method, employing identical input variables and sample data. The findings revealed that the RNN model displayed higher accuracy in comparison to the stepwise regression method^12^. Zhang et al.^13^ proposed a monthly temperature prediction model based on CEEMD–BiLSTM and applied it to the monthly temperature prediction of Zhengzhou City. Cai et al. predicted the monthly average temperature of Nanjing by using EMD combined with SVM. The intrinsic mode function (IMF) obtained after EMD decomposition can better reflect the physical properties of the system, but the EMD decomposition is sensitive to noise, so there are mode mixing and endpoint effects^14^.

From the information mentioned above, it is evident that both domestic and foreign researchers predominantly employ either a single model or a combination of multiple models for temperature prediction. In general, the coupled models tend to exhibit higher prediction accuracy compared to single-model approaches. Temperature data is a kind of time series data with obvious non-stationary fluctuation characteristics, so it is of rich theoretical and practical significance to establish a highly accurate and robust prediction model for temperature prediction. CEEMDAN model has the advantage of effectively extracting the time–frequency characteristics of nonlinear and non-stationary signals, BO algorithm has the advantage of quickly realizing optimization of the objective function within a limited number of iterations, and the BiLSTM modeling can effectively reveal the connection between current data and data from previous moments as well as data from future moments. The model effectively establishes a correlation between the current data and both the preceding and subsequent data, enabling a comprehensive analysis of temporal relationships. Therefore, the paper constructs a CEEMDAN–BO–BiLSTM model and applies it to the prediction of monthly average temperature in Jinan City, Shandong Province.

## Theory and methods

### Complete ensemble empirical mode decomposition (CEEMDAN)

The Complete Ensemble Empirical Mode Decomposition with Adaptive Noise (CEEMDAN) is an enhanced method built upon the EMD algorithm. It draws inspiration from the EEMD approach by incorporating Gaussian noise and iteratively averaging to counteract the effects of noise^15^. This method can better decompose the monthly rainfall time series data into several IMT components and residual components, and its calculation steps are as follows:

1. The rainfall time series and Gaussian white noise series are combined together to create a new rainfall time series. This new series is subsequently decomposed using EMD to obtain first-order intrinsic modal components (IMFs):
  $$im\overline{f}_{1} \left( t \right) = \frac{{\mathop \sum \nolimits_{i = 1}^{{\text{n}}} imf_{1}^{i} \left( t \right)}}{{\text{n}}}$$
  where $im{\overline{f} }_{1}(t)$ denotes the average component of the first-order IMT component^16^; $m{f}_{1}^{i}$ denotes the ith IMF component obtained after the first decomposition; and n denotes the maximum number of times white noise is added^17^.
2. Calculate the residuals of the first-order decomposition:
  $$x_{1} \left( t \right) = P\left( t \right) - im\overline{f}_{1} \left( t \right)$$
3. A Gaussian white noise sequence is introduced to the first-order residue ${x}_{1}(t)$ to generate the new decomposed sequence $x_{1}^{\prime } \left( t \right)$.This new sequence is then subjected to EDM decomposition to derive the second-order IMT component:
  $$im\overline{f}_{2} \left( t \right) = \frac{{\mathop \sum \nolimits_{i = 1}^{{\text{n}}} imf_{2}^{i} \left( t \right)}}{{\text{n}}}$$
4. Continue repeating the aforementioned steps until the residuals exhibit a monotonic behavior. At this stage, the rainfall time series is represented as:
  $${\text{P}}\left( {\text{t}} \right) = \mathop \sum \limits_{{{\text{j}} = 1}}^{{\text{m}}} im\overline{f}_{j} \left( t \right) + x_{{\text{k}}} \left( {\text{t}} \right)$$
  where ${x}_{{\text{k}}}({\text{t}})$ denotes the final residual.

### Bi-directional long short-term memory network (BiLSTM)

**Figure 1:**
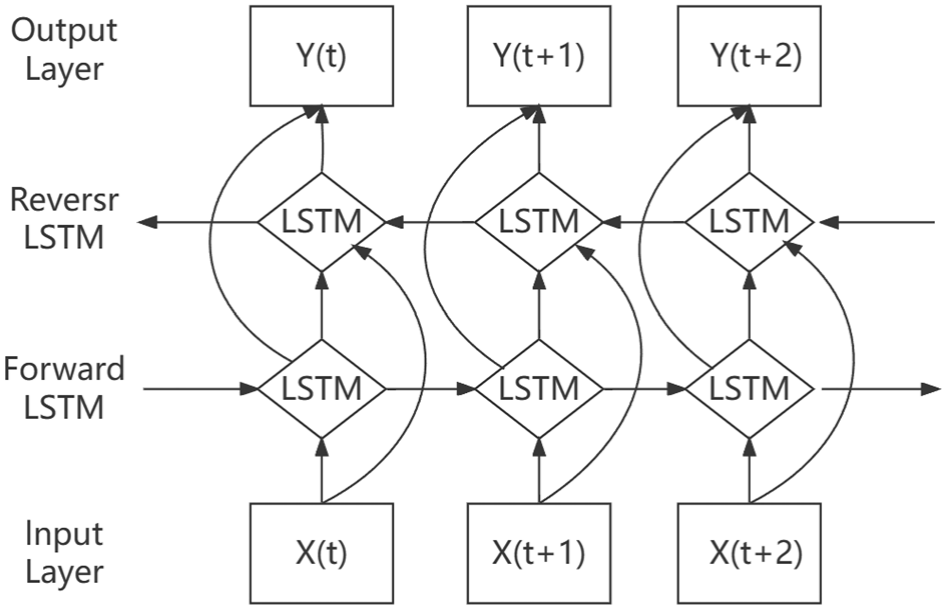
Bidirectional long and short-term memory neural network construction diagrams.

Bi-directional Long Short-Term Memory (BiLSTM) is to replace the ordinary RNN units in the traditional Bi-directional Recurrent Neural Network (BiRNN) with LSTM units, which is a combination of forward LSTM and backward LSTM^18^. The bi-directional network has the capability to learn sequential patterns by considering the inputs from both the start and end implicit layers of the sequence. This enables the exploration of connections between current data and previous as well as future moments, resulting in a more comprehensive analysis of inverse information, enhanced handling of long-term dependencies, and improved accuracy in model predictions^19^. The bi-directional long and short-term memory neural network construction is shown in Fig. 1.

### Bayesian optimization bidirectional long and short term memory network (BO–BiLSTM)

Bayesian (BO) is an optimization algorithm based on Bayesian statistics for finding the optimal solution within a limited number of iterations^20^. It selects the next sampling point for optimization by using prior information about the objective function and Bayesian inference. The steps of Bayesian optimization are as follows:

- *Step 1* Choose an agent model to represent the true function and establish its prior distribution.
- *Step 2* Given a set of observations, use Bayes’ rule to obtain the posterior.
- *Step 3* Apply the acquisition function to identify the subsequent sampling point.
  $$\begin{aligned} & X\_\left( {t + 1} \right) = argmax\left( {P\left( {f\left( x \right) \ge f\left( {x*} \right) + \varepsilon } \right)} \right) \\ & PI\left( X \right) = \emptyset \left( {\frac{{\mu_{t} \left( x \right) - f\left( {x^{ + } } \right) - \varepsilon }}{{\sigma_{t} \left( x \right)}}} \right) \\ \end{aligned}$$
- *Step 4* The newly sampled data is incorporated into the existing set of observations, following which step 2 is executed iteratively until convergence or the allocated budget is exhausted.

**Figure 2:**
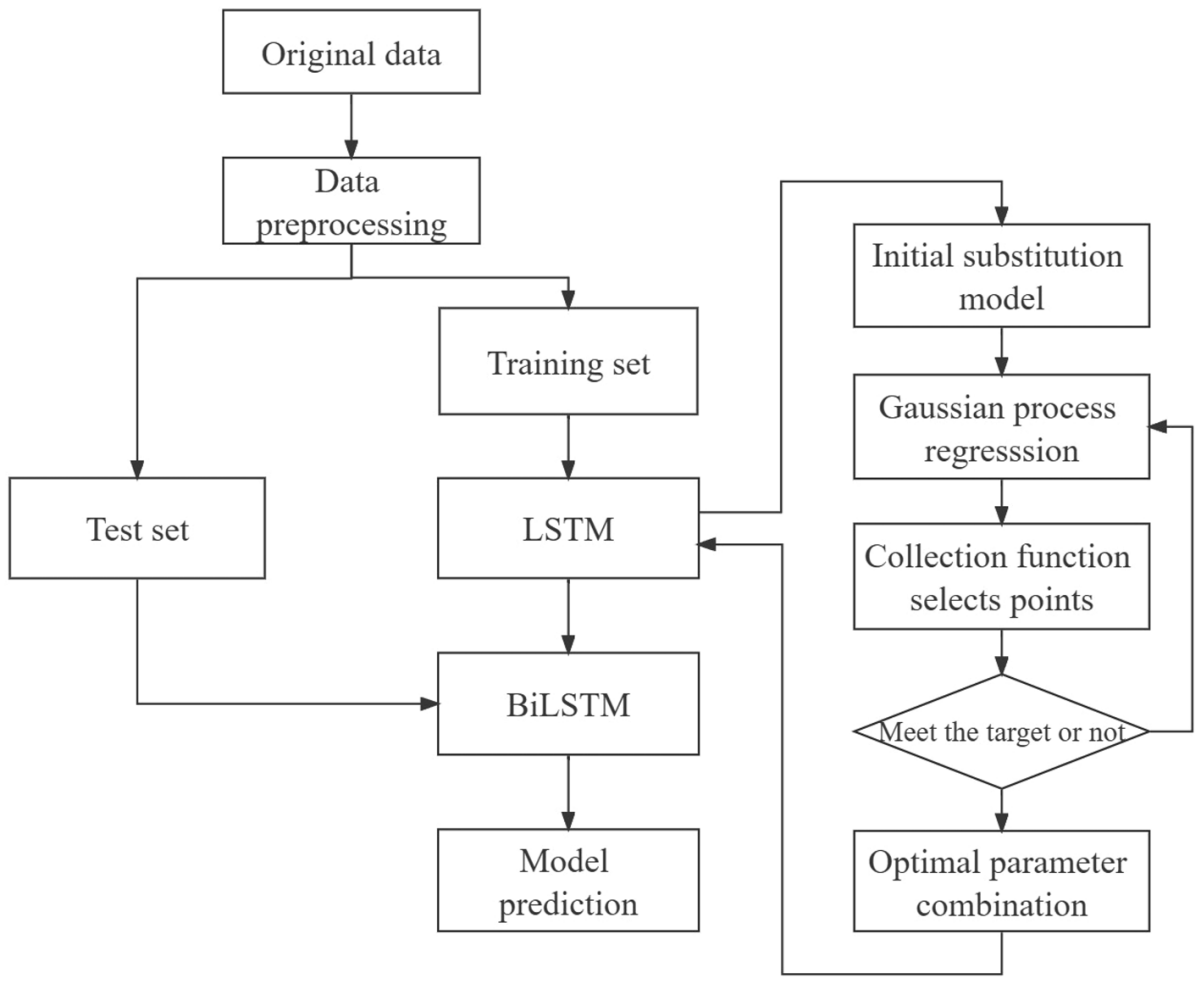
Flowchart of BO–BiLSTM algorithm.

The Bayesian optimization framework comprises two central components: the probabilistic agent model and the acquisition function. The probabilistic agent model encompasses both a priori probability model and an observation model^21^. The acquisition function is designed based on the posterior probability distribution function, The selection of suitable probabilistic agent model and acquisition function plays a crucial role in attaining improved optimization outcomes^22^. The flowchart of the algorithm for Bayesian optimization of bi-directional long and short-term memory network is shown in Fig. 2.

### Coupled CEEMDAN–BO–BiLSTM model prediction

**Figure 3:**
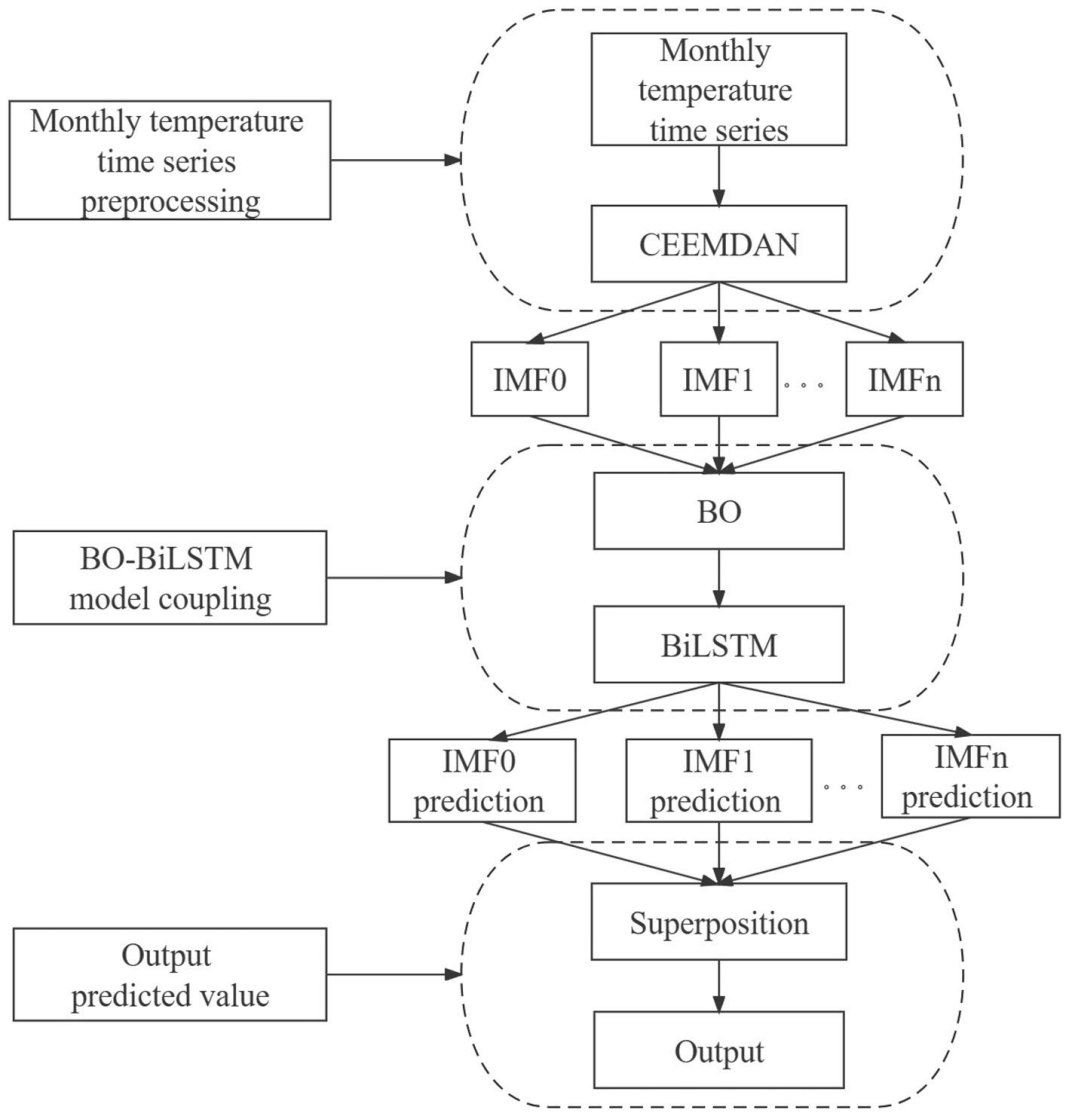
Flowchart of CEEMDAN–BO–BiLSTM model.

CEEMDAN, BO and BiLSTM are coupled into the CEEMDAN–BO–BiLSTM temperature prediction model with the following steps, and the flow chart of the CEEMDAN–BO–BiLSTM model is shown in Fig. 3.

- *Step 1* The model input consists of the average temperature information of n consecutive months;
- *Step 2* The original rainfall sequence is decomposed into k components using the CEEMDAN model;
- *Step 3* A Bayesian optimization algorithm coupled with a bidirectional long and short-term memory network (BO–BiLSTM) is developed;
- *Step 4* For each component, separate input of the BO–BiLSTM prediction model results in obtaining k individual prediction models;
- *Step 5* The predicted values of the k prediction models are summed correspondingly to obtain the predicted value of the average temperature of each month.

## Example applications

### Overview of the study area

**Figure 4:**
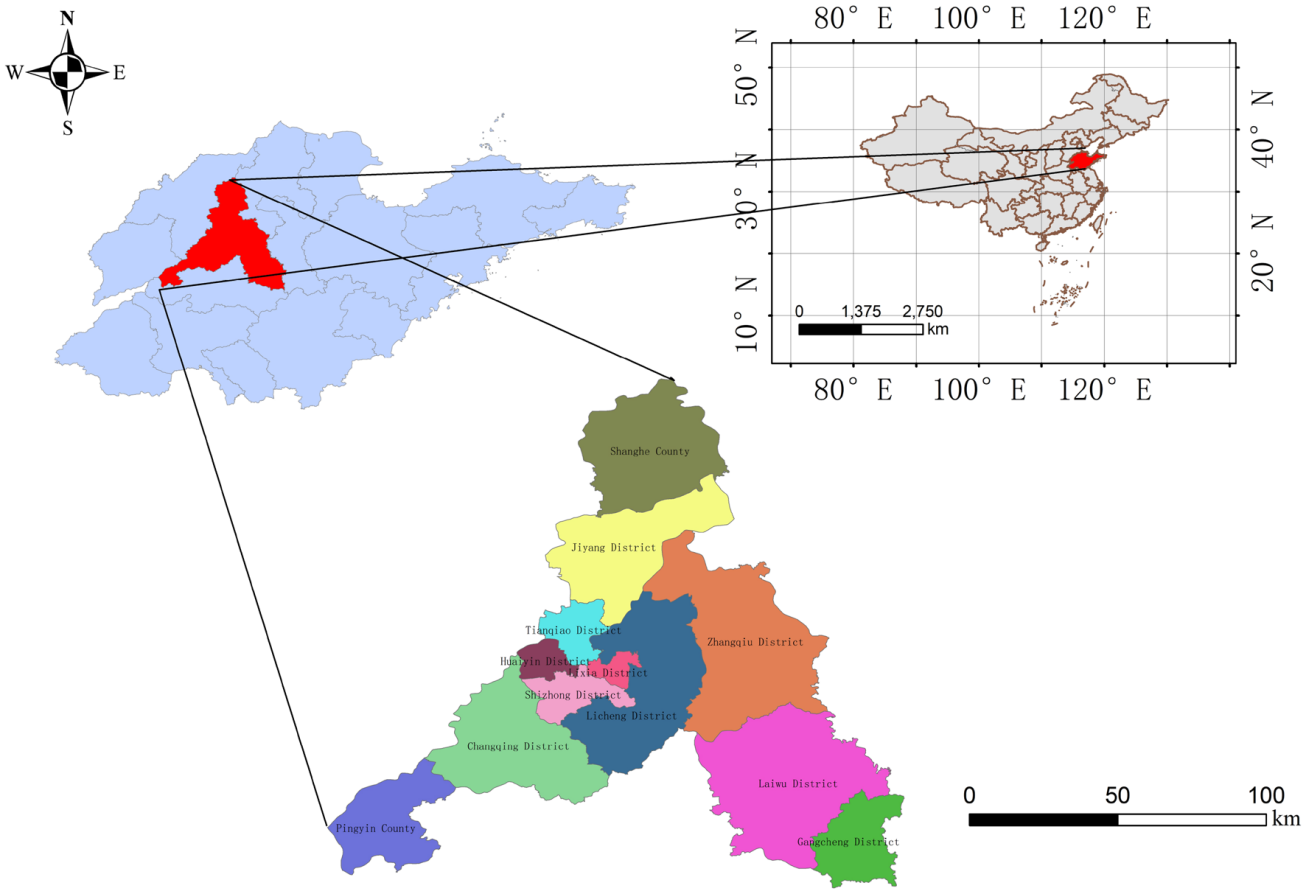
Regional Location Map of Jinan.

Jinan City is situated in the central west of Shandong Province, geographically located between latitude 36° 40′–37° 57′ north and longitude 116° 18′–117° 28′ east, adjacent to Mount Tai and west of the Yellow River, with a large east–west undulation and an overall northwest to southeast tilt. Jinan experiences a temperate monsoon climate characterized by hot and humid summers, as well as cold and dry winters^23^. Jinan City experiences significant temperature fluctuations, with an average annual temperature typically ranging between 14 and 15 °C. During the summer season, temperatures usually range from 24 to 28 °C, while in winter, they tend to hover around 0 °C. Temperatures in the spring and fall are pleasant for people to live in. The location map of the Jinan City is shown in Fig. 4, and this figure is created using ArcMap 10.2, URL:www.arcgis.com.

**Figure 5:**
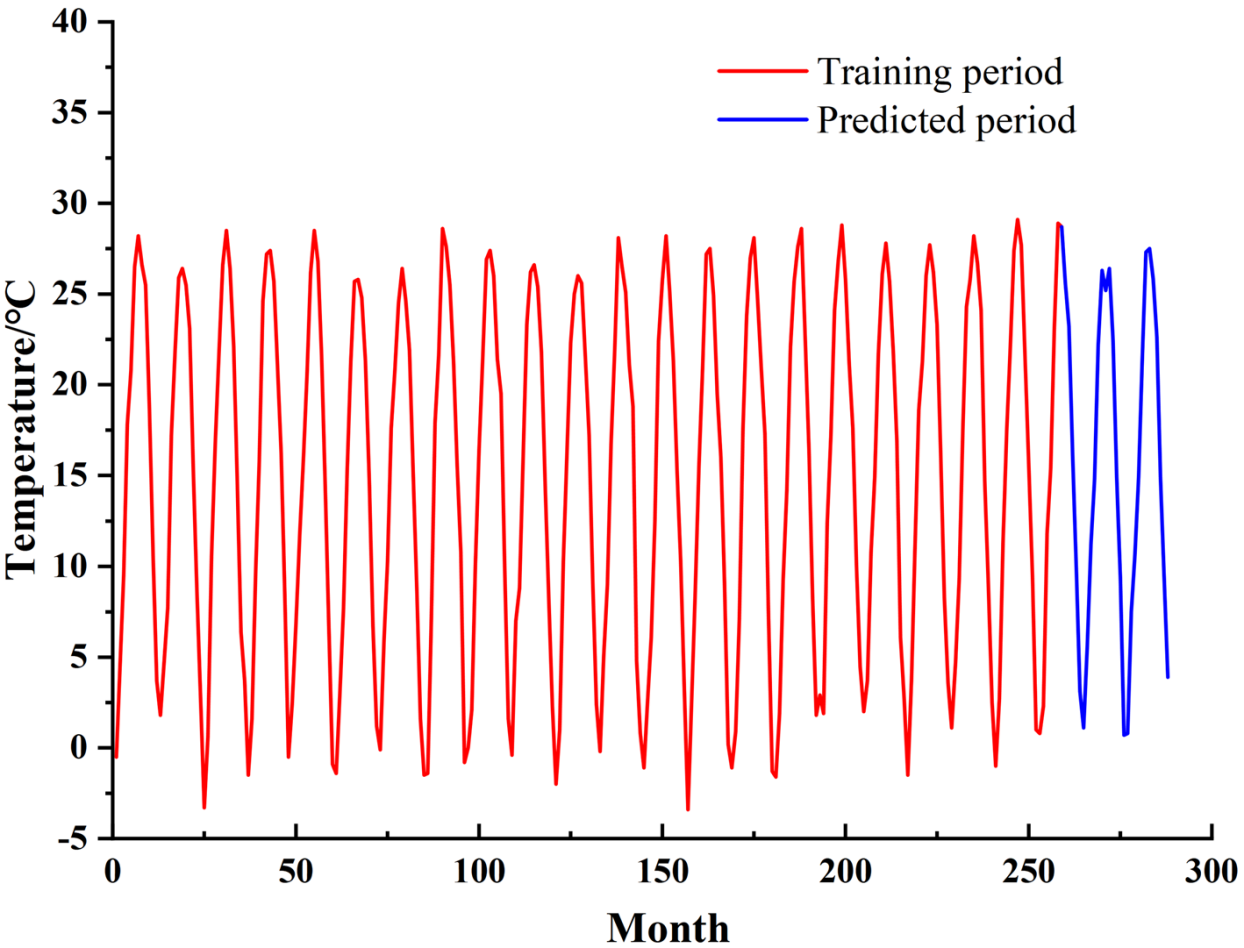
Time series plot of monthly mean air temperature.

In this study, the monthly mean temperature of Jinan City for 24 years from 1998 to 2021 is selected as the data source. The training set comprises the first 90% of the dataset, which is employed for temperature prediction testing. The remaining 10% of the dataset serves as the test set for temperature prediction evaluation. In addition, the raw temperature data of this study are all from the National Bureau of Statistics (http://www.stats.gov.cn/) to ensure the reliability and authenticity of the data source, and the software environment of this paper is Matlab 2020b.The time-series plot of the monthly average temperature in Jinan is shown in Fig. 5.

### Data decomposition

**Figure 6:**
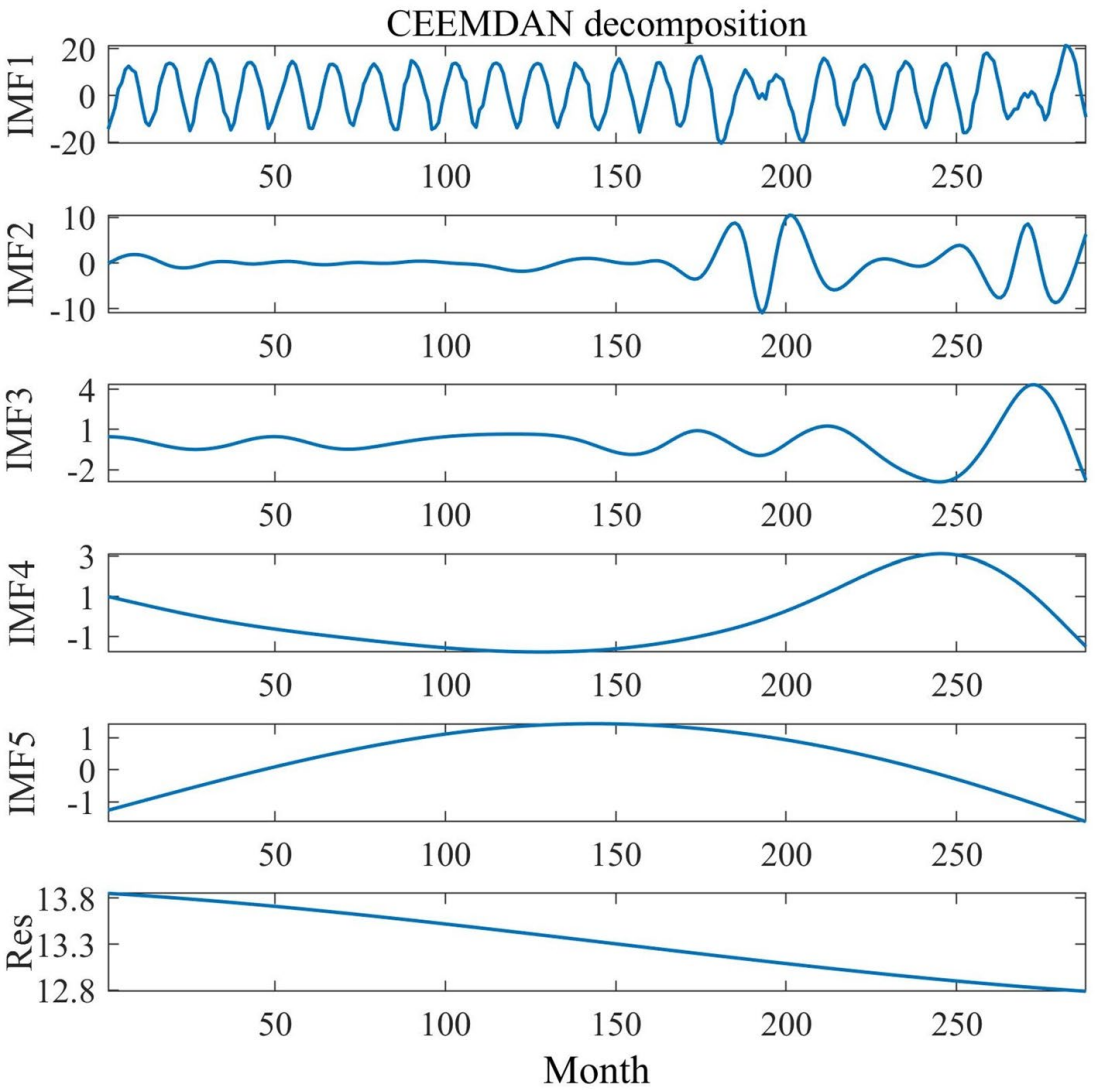
CEEMDAN decomposition.

The monthly mean temperature is subject to various factors like atmospheric circulation and seasonal variations. From the above figure, it is evident that the time series exhibits randomness and instability, indicating its characteristic as a nonlinear time series signal^24^. In this study, the MATLAB software component CEEMDAN model is utilized to decompose the monthly mean temperature time series, and five modal components and one residual component are finally obtained^25^. From the decomposition graph, it can be seen that the IMF1 component fluctuates more, and with the increase of decomposition times, the fluctuation and frequency of the IMF2 to IMF5 components are gradually reduced. From the residual value components, it can be seen that the temperature time series generally shows a decreasing trend. After decomposition, the time series of monthly average temperature has good smoothness. The CEEMDAN decomposition diagram is shown in Fig. 6.

### Prediction of monthly average temperature

**Figure 7:**
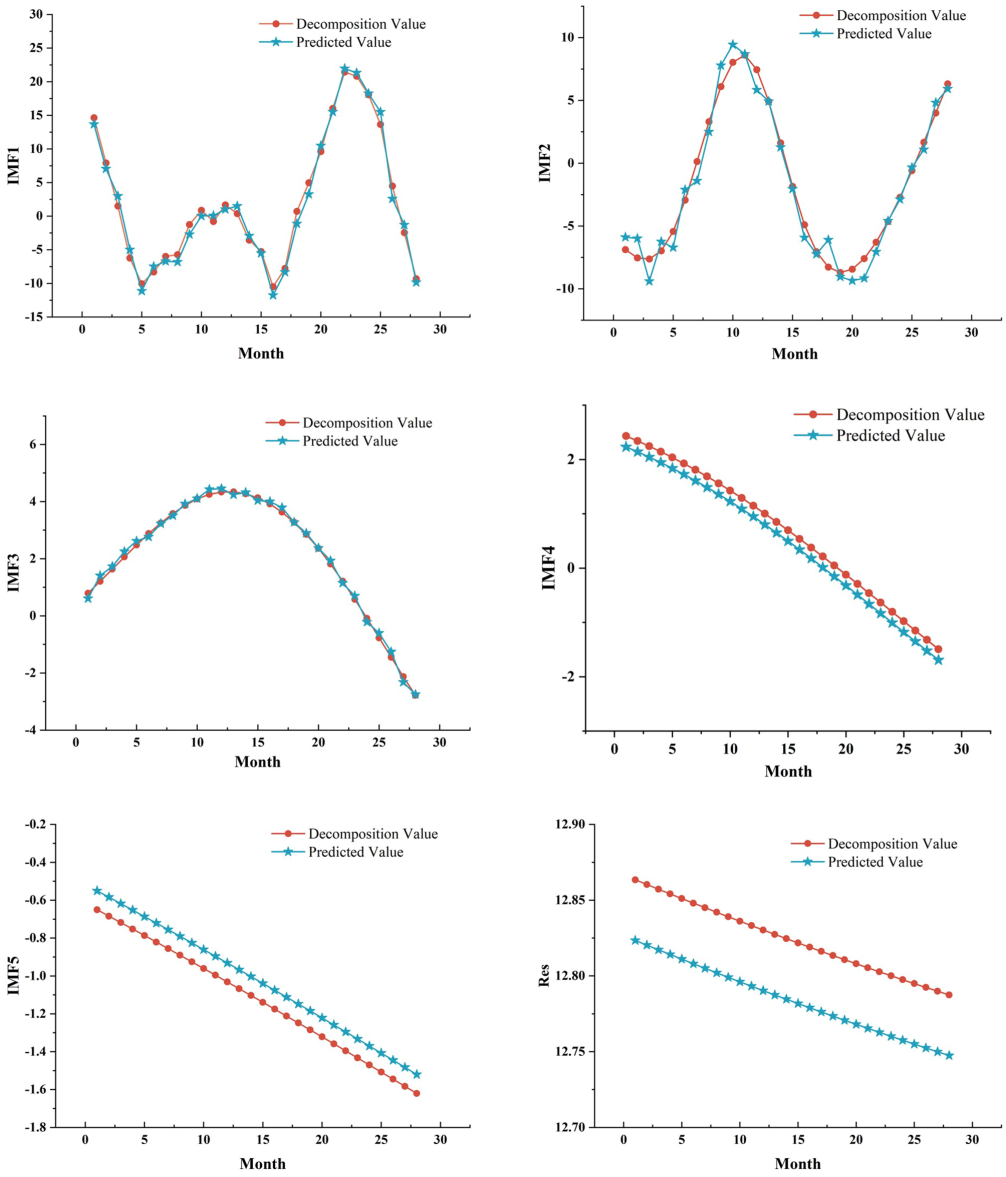
Plot of prediction results for each component.

The decomposition sequence (IMF1 to IMF5 and Res) of the monthly average temperature of Jinan City is used as a sample using the BiLSTM model after Bayesian optimization (BO), in which the first 90% of the data of each component (a total of 259 months) is used as a training set for temperature prediction. Rolling forecasts were used to produce the bottom 10% of the data, which was validated against the bottom 10% of the data for each component. The prediction results for each component are shown in Fig. 7.

**Figure 8:**
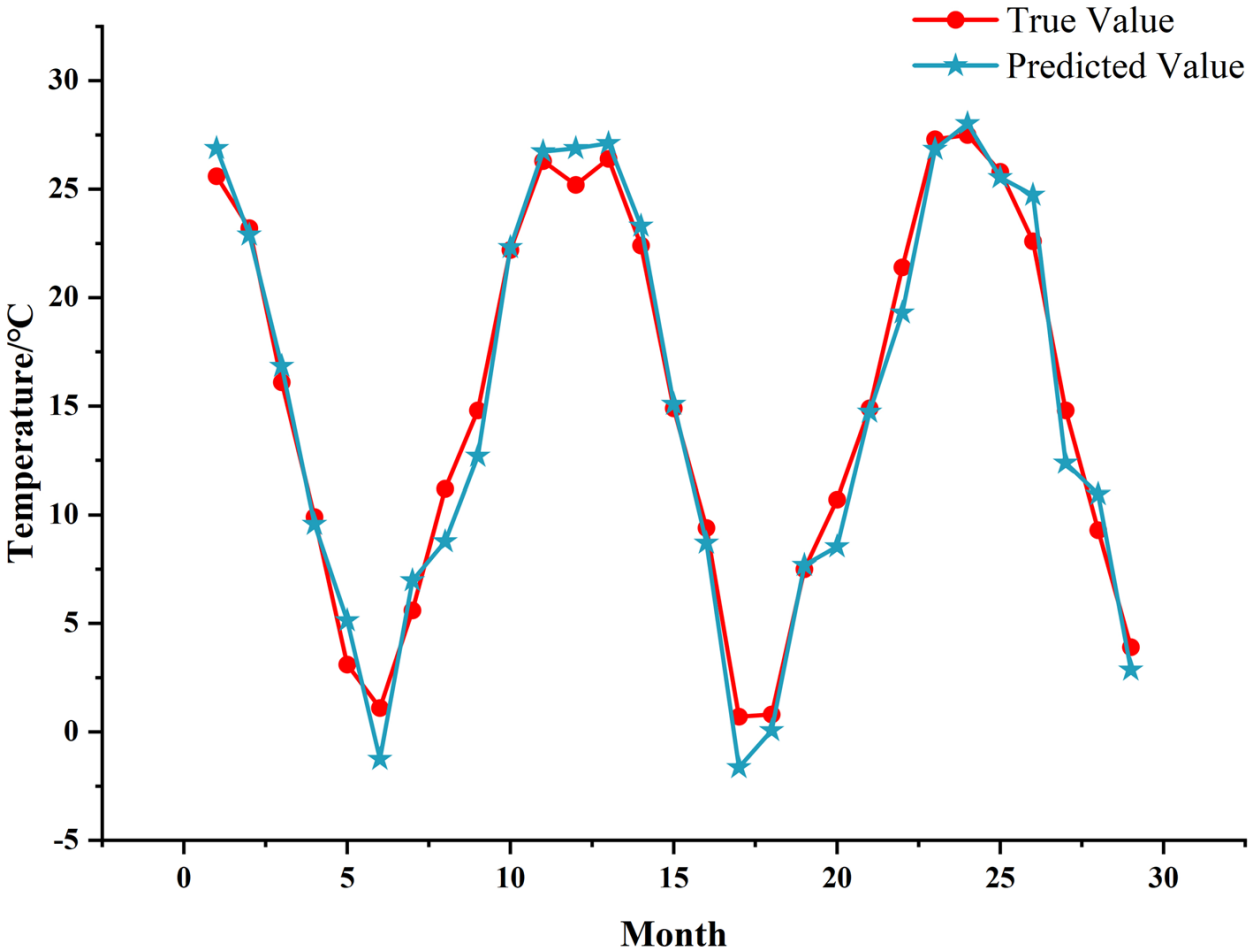
Comparison of forecast results.

As evident from Fig. 7, the BO-optimized BiLSTM prediction model predicts the components of the CEEMDAN decomposition with good prediction results. To provide a more intuitive view of the overall prediction performance, its overall prediction results are compared with all the data in the test set. The comparison chart of the prediction results is presented in Fig. 8. The relative error table of CEEMDAN–BO–BiLSTM model prediction is shown in Table 1

**Table 1:** Relative error table for CEEMDAN–BO–BiLSTM model prediction.

Month	True value	Predicted value	Relative error
1	25.6	26.9	0.050
2	23.2	22.9	0.013
3	16.1	16.8	0.046
4	9.9	9.6	0.033
5	3.1	5.1	0.655
6	1.1	− 1.3	2.144
7	5.6	7.0	0.247
8	11.2	8.8	0.216
9	14.8	12.7	0.142
10	22.2	22.3	0.005
11	26.3	26.7	0.017
12	25.2	26.9	0.067
13	26.4	27.1	0.027
14	22.4	23.3	0.040
15	14.9	15.1	0.013
16	9.4	8.7	0.073
17	0.7	− 1.6	3.343
18	0.8	0.1	0.915
19	7.5	7.7	0.025
20	10.7	8.5	0.201
21	14.9	14.7	0.011
22	21.4	19.3	0.098
23	27.3	26.9	0.016
24	27.5	28.0	0.019
25	25.8	25.5	0.010
26	22.6	24.7	0.094
27	14.8	12.4	0.164
28	9.3	10.9	0.177
29	3.9	2.9	0.267

As evidenced by Fig. 8 and the above table, in the prediction of monthly average temperature by CEEMDAN–BO–BiLSTM model, the real and predicted values have a good fitting effect, and most of the rest of the data basically coincide with each other, except for a small amount of error between the prediction results of individual months and the actual values. The minimum error is 0.005, the maximum error is 3.343, and the error is within 5%, which indicates that the model exhibits a higher level of prediction accuracy, reflecting that the model is somewhat reasonable.

## Discussion

To further evaluate the prediction effectiveness of the CEEMDAN–BO–BiLSTM model, In this study, a comparison is made between the prediction results of the CEEMDAN–BO–BiLSTM model and the three other models: CEEMDAN–BiLSTM, EMD–BiLSTM, and BiLSTM. To accurately assess the performance of the four prediction models, evaluation metrics including Root Mean Square Error (RMSE), Mean Absolute Error (MAE), Mean Absolute Percentage Error (MAPE) are utilized. The evaluation metrics of the four models are presented in Table 2, and their formulas are as follows:

$${\text{RMSE}} = \sqrt {\frac{1}{n}\mathop \sum \limits_{i = 1}^{n} \left( {{\text{P}}_{i} - {\text{P}}_{i}^{*} } \right)^{2} }$$

$${\text{MAE}} = \frac{1}{n}\mathop \sum \limits_{i = 1}^{n} \left| {{\text{P}}_{i} - {\text{P}}_{i}^{*} } \right|$$

$${\text{MAPE}} = \frac{1}{n}\mathop \sum \limits_{i = 1}^{n} \left| {\frac{{{\text{P}}_{i} - {\text{P}}_{i}^{*} }}{{{\text{P}}_{i} }}} \right|$$

**Table 2:** Table of indicators for the four forecasting models.

Model	MAE	RMSE	MAPE
CEEMDAN–BO–BiLSTM	1.17	1.43	0.31
CEEMDAN–BiLSTM	2.59	2.88	0.54
EMD–BiLSTM	3.24	3.62	0.67
BiLSTM	5.13	5.69	1.13

Based on the table provided, it is evident that the constructed CEEMDAN–BO–BiLSTM-based model shows better prediction performance, and its root mean square error is reduced by 1.45, 2.19 and 44.26, respectively, compared to the other three models. In a word, all kinds of error indexes of the CEEMDAN–BO–BiLSTM model are lower than the other three models. The primary factor behind this disparity is that the CEEMDAN model can better adapt to the nonlinear and nonsmooth characteristics of the signal, while The BO optimization algorithm proves to be a versatile tool for analyzing, predicting, and extracting features from temperature time series data due to its flexibility. Further, The single BiLSTM model exhibits the lowest prediction performance, indicating that the overall predictive capability of a single model is inferior to that of the coupled model.

In addition to the intuitive error metrics described above, we also compared the performance of the average errors using the Friedman test. Meanwhile, we calculated the average running time by recording 10 random runs of each model, as shown in Table 3, and the results of the Friedman test are recorded in Table 4.

**Table 3:** Average time for each model run.

Model	CEEMDAN–BO–BiLSTM	CEEMDAN–BiLSTM	EMD–BiLSTM	BiLSTM
Average elapsed time	82.42	76.26	78.65	46.87

**Table 4:** Nonparametric test results.

	Model	Ordinal mean
Friedman test	Original value	3.38
	CEEMDAN–BO–BiLSTM	3.32
	CEEMDAN–BiLSTM	2.81
	EMD–BiLSTM	2.83
	BiLSTM	2.64

**Figure 9:**
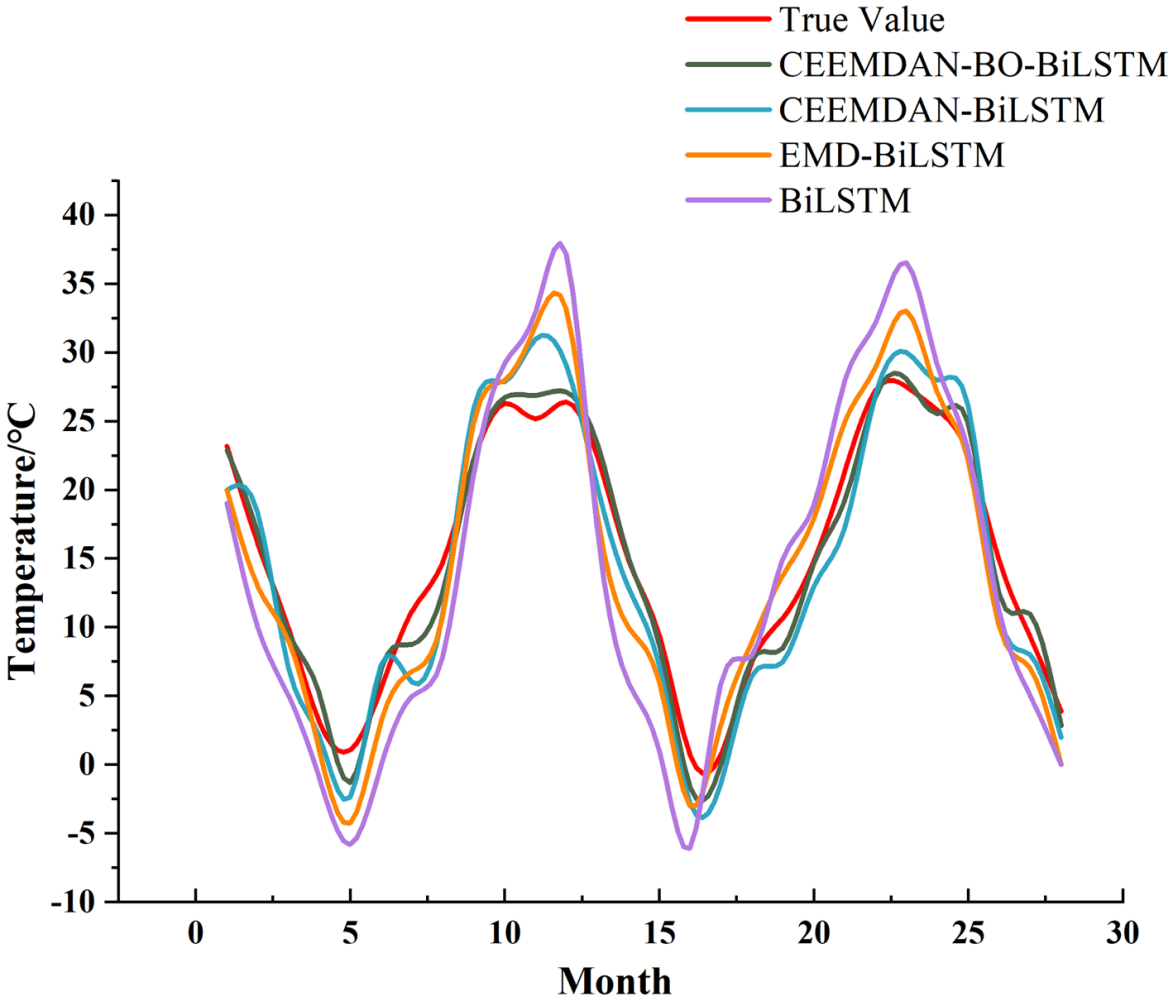
Plot of the prediction results of the four models.

**Figure 10:**
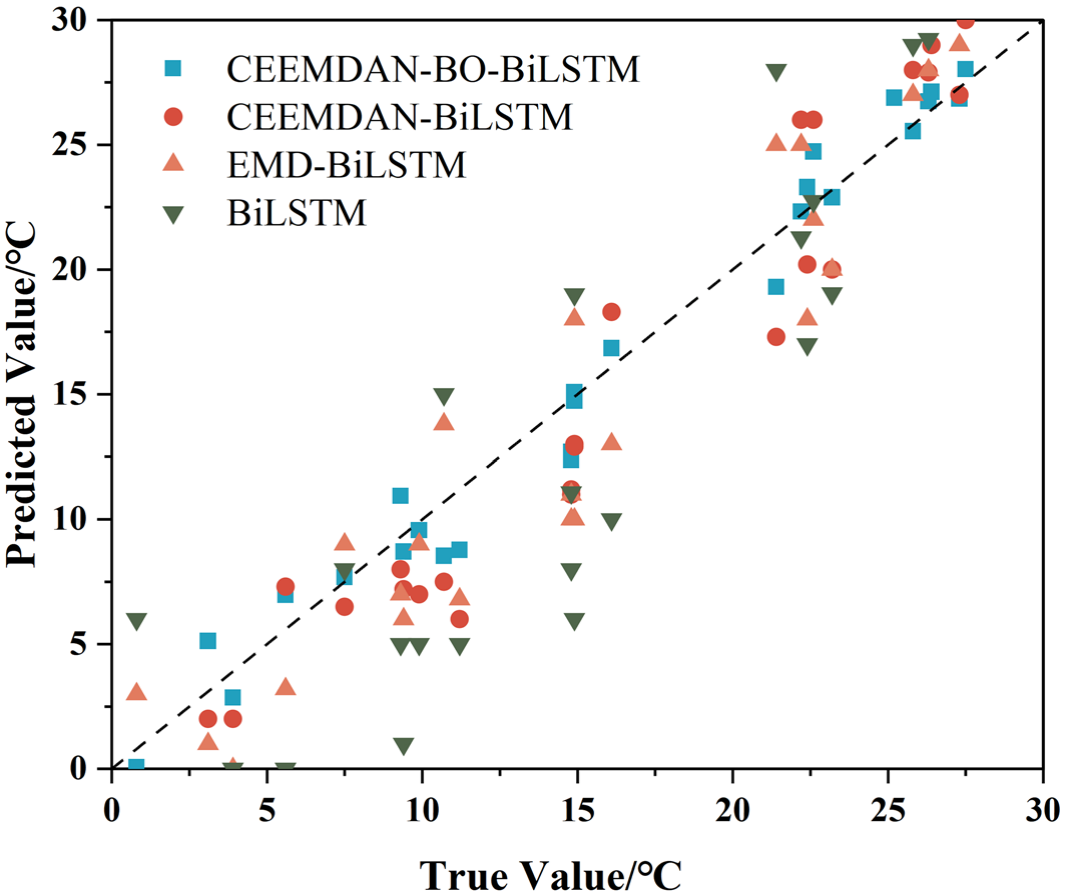
Scatterplot of the prediction results of the four models.

**Figure 11:**
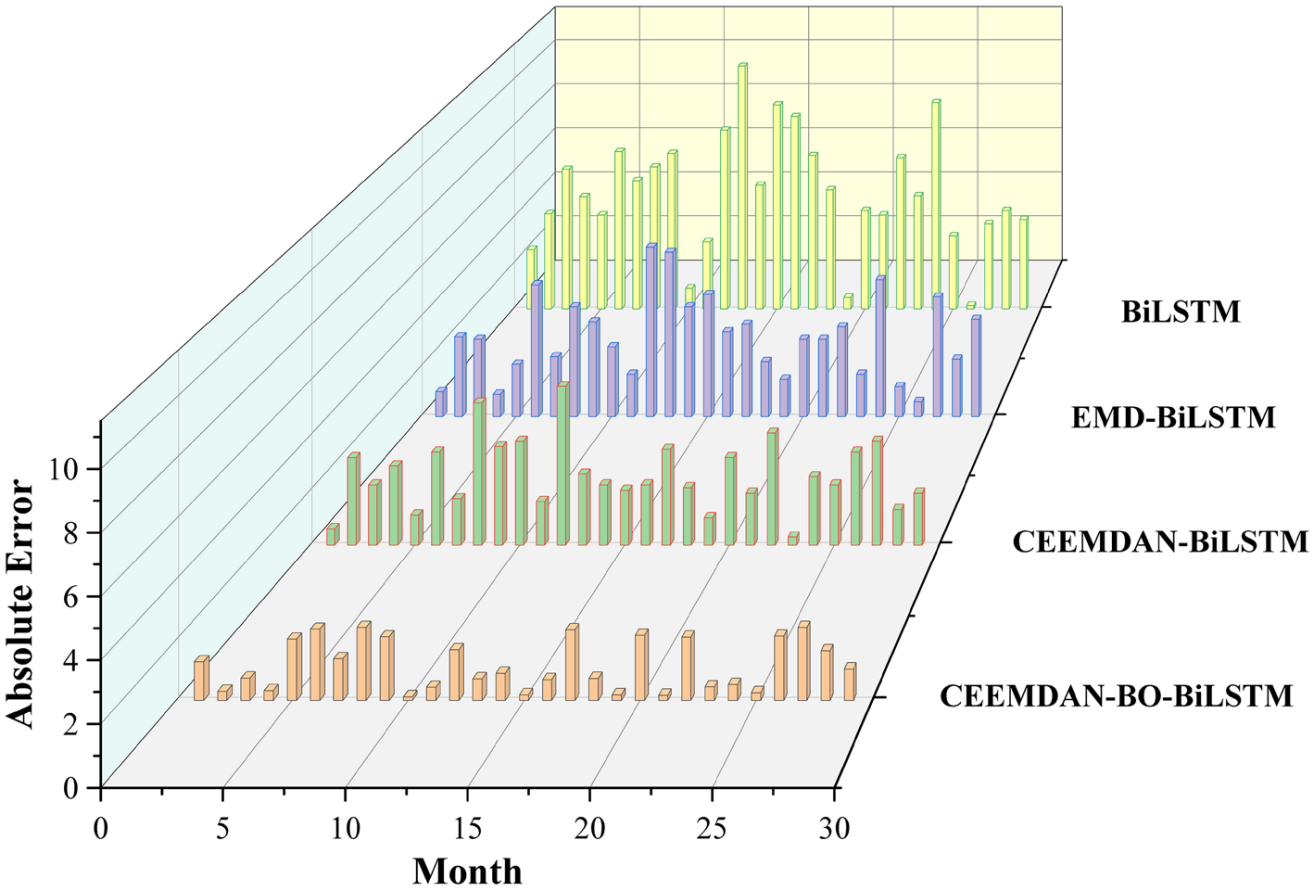
Comparison of absolute errors in the prediction results of the four models.

In order to observe the prediction performance more intuitively, the prediction results of the four models are plotted, and the curves are shown in Fig. 9, the scatter plot is shown in Fig. 10, and the comparison of absolute errors is shown in Fig. 11.

The various comparison plots of the above four models indicate that the CEEMDAN–BO–BiLSTM model has a strong temperature prediction capability. The single BiLSTM model is the worst predictor among the four models, and the prediction performance of the coupled model is generally higher than that of the single prediction model. This may be due to the fact that there are more factors affecting the temperature, and a single time series cannot fully consider the influence mechanism of temperature. CEEMDAN is a signal processing method based on the EMD decomposition improved, which is able to adaptively decompose the signal and carry out the noise adaptive processing on each residual component, which makes it able to better adapt to the nonlinear and nonsmooth characteristics of the signal, and greatly improves the accuracy and stability of the decomposition. Accuracy and stability. After the data is decomposed by CEEMDAN model, the parameters and learning process of BiLSTM prediction model are improved with the help of BO model optimization technique, and the coupled CEEMDAN–BO–BiLSTM model has better prediction performance and higher prediction accuracy.

EMD is a data decomposition technique that is adaptive in nature, which was originally introduced by Huang et al. in 1998 for decomposing a non-smooth signal into a set of eigenmode functions, but there are problems such as mode mixing and boundary effects in practice. Based on these problems, some scholars proposed the CEEMD method to reduce mode aliasing and boundary effects by adding Gaussian white noise to improve the quality and stability of the decomposition results. Further, Torres et al. proposed the CEEMDAN method in 2011, which introduces adaptive noise on the basis of CEEMD by replacing the Gaussian white noise with the noise of the signal itself and adaptively adjusting the noise intensity to better adapt to the characteristics of the signal. The CEEMDAN–BiLSTM model can be seen from the four comparative figures in Figs. 9, 10, and 11, which show that the CEEMDAN–BiLSTM model has a better prediction effect than the EMD–BiLSTM model.

In conclusion, in the comparison of the performance of the four models, CEEMDAN–BO–BiLSTM, CEEMDAN–BiLSTM, EMD–BiLSTM, and BiLSTM, it is seen that the CEEMDAN decomposition method adds the adaptive intensity of the noise of the signal itself, which is able to better deal with the nonlinear and non-stationary temperature signals, and further improves the performance of the decomposition; BO algorithm further improves the decomposition performance by adding the adaptive intensity of the noise of the signal itself within an effective number of iterations to quickly find the optimal solution region, and at the same time can deal with the noise and inconductance of the temperature function, which is characterized by high efficiency and flexibility; furthermore, the BiLSTM model is able to capture the information of the before and after series of temperatures, which is more conducive to the prediction of the nonsmooth and nonlinear temperature time series. Considering the advantages of these three models, the constructed CEEMDAN–BO–BiLSTM model is feasible in predicting nonlinear temperature series with better prediction performance and higher accuracy.

## Conclusion

1. The CEEMDAN model is a method for decomposing signals that offers several benefits, including adaptability, robustness, effective noise suppression, and efficient computation. The BO model is an optimization method based on Bayesian statistics and Gaussian process, and the algorithm has been specifically modified or adjusted to handle and analyze temperature-related data. The BiLSTM model has the advantages of powerful modeling capabilities, bi-directional information flow, high accuracy and robustness^26^. Considering the advantages of these three models, to highlight their respective strengths, this paper constructs the CEEMDAN–BO–BiLSTM model and applies it to the prediction of monthly average temperature in Jinan City, Shandong Province. The results indicate that the model has a certain degree of feasibility in predicting nonlinear and non-stationary temperature series.
2. In comparison to the other three forecasting models, the constructed CEEMDAN–BO–BiLSTM model has a root mean square error of 1.43, an average absolute error of 1.17, an average absolute percentage error of 0.31%, with the smallest value of each error and the largest coefficient of determination. The results indicate that the CEEMDAN–BO–BiLSTM model has a good performance in predicting temperature series.
3. The temperature prediction of Jinan City, Shandong Province in this paper needs to be improved, the temperature series are affected by various factors such as atmospheric circulation, monsoon climate, etc., and the BO optimization algorithm has the problems of complicated parameter setting and high computational cost. The next step is to focus on the algorithm modification for the study area and the prediction of time series with different characteristics.

## Data Availability

Data and materials are available from the corresponding author upon request.
